# Dissecting immunological mechanisms underlying influenza viral nucleoprotein-induced mucosal immunity against diverse viral strains

**DOI:** 10.1080/22221751.2024.2427792

**Published:** 2024-11-07

**Authors:** Wanyue Zhang, Angela Sloan, Jérémie Prévost, Levi Tamming, Sathya Raman, Annabelle Pfeifle, Caroline Gravel, Wangxue Chen, Anwar M. Hashem, Jianguo Wu, Jingxin Cao, Michael J. W. Johnston, Lisheng Wang, Simon Sauve, Michael Rosu-Myles, Darwyn Kobasa, David Safronetz, Xuguang Li

**Affiliations:** a Centre for Biologics Evaluation, Biologic and Radiopharmaceutical Drugs Directorate, HPFB, Health Canada and WHO Collaborating Center for Standardization and Evaluation of Biologicals, Ottawa, Canada; b Department of Biochemistry, Microbiology and Immunology, Faculty of Medicine, University of Ottawa, Ottawa, Canada; c National Microbiology Laboratory, Public Health Agency of Canada, Winnipeg, Canada; d Human Health Therapeutics Research Center, National Research Council of Canada, Ottawa, Canada; e Vaccines and Immunotherapy Unit, King Fahd Medical Research Center, King Abdulaziz University, Jeddah, Saudi Arabia; f Department of Clinical Microbiology and Immunology, Faculty of Medicine, King Abdulaziz University, Jeddah, Saudi Arabia; g Department of Chemistry, Carlton University, Ottawa, Canada

**Keywords:** Influenza, nucleoprotein, universal vaccine, H5N1, intranasal, intramuscular

## Abstract

The nucleoprotein (NP) of type A influenza virus (IAV) is highly conserved across all virus strains, making it an attractive candidate antigen for universal vaccines. While various studies have explored NP-induced mucosal immunity, here we interrogated the mechanistic differences between intramuscular (IM) and intranasal (IN) delivery of a recombinant adenovirus carrying NP fused with a bifunctional CD40 ligand. Despite being less effective than IM delivery in inducing systemic cellular immune responses and antibody-dependent cellular cytotoxicity (ADCC), IN immunization elicited superior antigen-specific recall humoral and cellular response in the nasal associated lymphoid tissue (NALT) of the upper respiratory tract, the initial site of immune recognition and elimination of inhaled pathogens. IN vaccination also induced significantly stronger pulmonary T cell responses in the lower respiratory tract than IM vaccination, in particular the CD8 T cells. Moreover, blocking lymphocyte circulation abrogated IM but not IN immunization induced protection, illustrating the critical role of local memory immune response upon viral infection. Notably, the CD40-targeted nasal delivery not only improved the magnitude but also the breadth of protection, including against lethal challenge with a newly isolated highly pathogenic avian H5N1 strain. These findings are informative for the design of universal mucosal vaccines, where the predominant mode of protection is independent of neutralizing antibodies.

## Introduction

Influenza is a highly contagious respiratory virus that has caused more than six distinct pandemics and several epidemics in the past century. Every year, seasonal flu causes an estimated one billion infections and results in 290,000–650,000 deaths worldwide [1]. The year-to-year efficacy of licenced vaccines is highly variable, ranging from 19% to 60% [2]. Influenza vaccine efficacy is affected by a wide range of factors, such as mismatches between predicted vaccine strains and the circulating strains, a problem exacerbated by the wide range of animal reservoirs that can lead to reassortment [3]. With the ongoing panzootic event caused by the highly pathogenic avian influenza (HPAI) and the rising number of cases in the recent flu seasons, there is an urgent need to understand and develop universal influenza vaccines that induce broadly protective immunity at the mucosal surface [2, 4].

Despite emerging evidence showing enhanced vaccine efficacy via intranasal (IN) administration, routine influenza vaccination remains intramuscular (IM). The only available IN influenza vaccine, live attenuated influenza vaccine (LAIV), is less effective in adults than children and is only recommended for healthy populations aged 2–59 [5–7]. Furthermore, human studies rarely evaluate the underlying mechanisms of protection induced by IN vaccination due to technical limitations of tissue sampling at mucosal sites.

While the current influenza vaccines mainly target surface proteins, such as hemagglutinin (HA), the nucleoprotein (NP) remains another desirable antigen due to its abundance in infected cells and its highly conserved sequence. Unlike the surface antigens, which are under constant selective pressure exerted by neutralizing antibodies, NP is highly conserved across all influenza A subtypes. This high conservation is also due to the critical and multifunctional role of NP in RNA packaging, nuclear trafficking, and viral RNA transcription and replication [8–10]. Over the last several decades, extensive studies have explored the potential of NP as a vaccine antigen, yielding mixed results. Specifically, NP has been studied in various platforms, such as viral-vectored vaccines [11–15], protein subunit or peptide vaccines [16–20] and mRNA vaccines [21–25]. Additionally, various T cell-based influenza vaccines were tested in clinical trials, including self-assembling nanoparticles targeting the NP [18], peptide-based constructs targeting multiple epitopes [26–28], and a Modified vaccinia Ankara (MVA) – vectored construct expressing NP and M1 [29]. The parenteral administration of these vaccines successfully induced significant antigen-specific T cell responses in peripheral blood. However, in a controlled human infection challenge model testing the MVA-vectored vaccine, the immunization had no effect on the nasopharyngeal viral load upon infection. This suggests that the peripheral T cells may not be sufficient to provide effective protection [29], which supports observations from our study. Better induction of mucosal immunity would be desirable, highlighting the necessity of understanding the mechanism of IN vaccination [30, 31]. Notably, several influenza vaccine studies have demonstrated that IN administration provides better protection than IM by generating strong immune responses in the lungs [11, 32–37]. Yet, more in-depth studies are needed to compare the immune responses elicited by the two routes of administration, particularly at addressing both systemic and mucosal immunities.

In this study we aim to examine the IN and IM administration of a CD40 ligand (CD40L)-adjuvanted NP vaccine. CD40L has been employed by various labs as an adjuvant, demonstrating robust protection [38–42]. It simultaneously acts as a targeting ligand to promote uptake by antigen-presenting cells (APCs) and as a molecular adjuvant that stimulates APC activation [43]. Therefore, the ectodomain of mouse CD40L was fused to the NP sequence to enhance immunogenicity and to sustain an effective immune response. Thus, in this study we used the vaccine construct, Ad-NP-CD40L, to interrogate the differences in protective mechanisms elicited by IN and IM administration. The comparison focused on less-studied aspects, such as characterization of systemic and mucosal immunities and the memory responses in the nasal-associated lymphoid tissue (NALT), especially in the context of eliciting cross-subtype protection upon exposure to diverse strains of viruses, including a newly isolated highly pathogenic avian influenza (HPAI) H5N1**.**

## Methods

### Mice

Six-week-old female BALB/c mice (Charles River) were used for all animal experiments. All animal procedures were performed in accordance with institutional guidelines and ethical approval was granted by the Animal Care Committee at Health Canada, Ottawa, ON, Canada and the Public Health Agency of Canada, Winnipeg, MB, Canada. Animal experiments were performed under Animal Utilization Protocol (AUP) H21-019, 2021-011, 2022-007, and 2023-004.

### Generation of rAds

Recombinant adenoviruses constructs (rAds) were generated as previously described [32]. In brief, the Ad-NP-CD40L construct was designed to express a trimeric, secreted form of influenza A/duck/Yokohama/aq10/03 (H5N1) NP (GenBank accession #AB212281), with 23 amino acids from the human tyrosinase signal peptide (GenBank accession # AH003020) at the N terminus, fused to a 27 amino acid fragment from the bacteriophage T4 fibritin trimerization motif connected to the ectodomain of mouse CD40L (GenBank accession #NM_011616, aa 117–260) (Supp Figure 1). Empty vector control was used as controls. rAds were generated using AdenoVator Adenoviral Expression System with pAdenoVator-CMV5 (Cuo)-IRES-GFP transfer vector (Qbiogene, Carlsbad, CA) according to the manufacturer’s instructions. Cloning was confirmed by DNA sequencing and restriction enzyme digestion. For vaccination, the rAds constructs were amplified in HEK-293A cells and purified by ultracentrifugation with a 30% sucrose cushion. rAd stocks were titrated using the Adeno-X Rapid Titer Kit (Takara Bio USA Inc.).

### Weight loss and survival studies

Mice were immunized intranasally or intramuscularly with 10^9^ PFU of each rAd construct in 25 μl or 50 μl, respectively. Mice were prime immunized on day 0 and boosted on day 28. Four weeks post-boost vaccination, mice were challenged intranasally with 1000 PFU of the A/Netherlands/602/09 (H1N1), 3.85 × 10^5^ PFU of A/Hong Kong/01/68(H3N2), or 10 PFU of A/ RT.Hawk/ON/2022 (H5N1) influenza virus in 25 μl. The mice were weighed and monitored for signs of illness for 14 days post-challenge. A separate group of mice were sacrificed at the peak of illness for viral load determination. Five days post-challenge for A/Netherlands/602/09 (H1N1), 3 days for A/Hong Kong/01/68(H3N2), and 4 days for A/ RT.Hawk/ON/2022 (H5N1); necropsy days determined by previous preliminary challenge experiments. Lung and nasal turbinates were collected from 4 or 5 mice per group for viral load determination.

### Tissue collection

Serum was collected from vaccinated mice 21 days after prime vaccination (Day 21) and 21 days after boost (Day 49) for measurement of antibody levels and antibody-dependent cellular cytotoxicity. Bronchoalveolar lavage fluid (BALF) was collected on 4 weeks post-boost (Day 56) for mucosal antibodies analysis. Spleens were collected 4 weeks post-boost (Day 56) for systemic cytokine level measurement. Nasal-associated lymphoid tissue (NALT) was collected on Day 61, 5 days after an intranasal challenge of 1000 PFU of influenza A/Netherlands/602/09 (H1N1). The tissue was isolated and cultured as previously described [44]. Briefly, after removing NALT from the upper palate of the sacrificed mouse, it was successively washed eight times in RPMI 1640 with 10% FBS. The NALT was then transferred into a new 48-well plate containing the same media and cultured in a 5% CO2 incubator at 37 °C for 24 hours. The supernatant was collected to determine mucosal antibody and cytokine levels.

### Viral titration

The plaque assay was performed for the H1N1 challenge as described previously [32]. Briefly, the lungs and nasal tissues were harvested 5 days post challenge and flash frozen in liquid nitrogen. Following thawing on ice, lungs and nasal tissues were homogenized with a pestle in 300μl and 250μl of PBS, respectively. After centrifugation and filtration with a 0.45um syringe filter, ten-fold serial dilutions of the homogenates were prepared in serum-free complete DMEM medium supplemented with 25 mM HEPES buffer, 0.2%BSA and 2 μg/ml TPCK-treated trypsin. The homogenate inoculums incubated on confluent MDCK cells for 2 hours, at 37°C. After removing the inoculum, cells were washed and overlaid with complete DMEM medium containing 25 mM HEPES buffer, 0.2% BSA, 2 μg/ml TPCK-treated trypsin and 0.8% agarose. After incubation for 4 days at 37°C/5% CO2, the cell monolayers were stained with crystal violet for plaque counting.

TCID_50_ assays were performed for H3N2 and H5N1 challenges as previously described [45]. Lungs and nasal turbinates were weighed and homogenized in 1 mL of MEM – 0.1% BSA – L-Glu – 2 penicillin–streptomycin (PS) with a 5-mm stainless steel bead using a Bead Ruptor Elite tissue homogenizer (Omni). Cell debris was removed by centrifugation, leaving the supernatant for viral load detection. Samples were serially diluted (1:10) in MEM supplemented with 0.1% BSA, L-Glu, and TPCK-trypsin (MEM – BSA – L-Glu – trypsin). 100 μL of each dilution was added to the wells of a 96-well plate of MDCK cells in triplicate, followed by incubation at 37°C with 5% CO2 for 4–5 days. The presence of cytopathic effect (CPE) was observed and the TCID_50_ titer per millilitre or gram of tissue was determined using the Reed-Muench method [46].

### Antibody detection by enzyme-linked immunosorbent assay (ELISA)

The end-point titers of serum, NALT supernatant, and BALF anti-NP antibodies were determined as described previously, with minor adjustments [25]. Briefly, 96-well plates were coated with 100 μl /well of 0.5 μg/ml of recombinant influenza A H1N1 (A/California/07/2009) nucleoprotein (Sino Biological Inc., 40205-V08B). Overnight incubation at 4°C was followed with PBS wash containing 0.05% Tween 20 (PBS-0.05 T) and blocked for 2 hours at 37°C with 3% BSA in PBS-0.05 T. After another wash, two-fold serial dilutions of the serum, NALT supernatant, or BALF in blocking buffer were added for 1 hour at 37°C. Following a wash, antibody binding was detected by HRP-conjugated anti-mouse IgG (Cytiva), anti-mouse IgG1, IgG2a, IgG2b (Jackson Immunoresearch Laboratories) or anti-mouse IgA (Life Technologies). The plates were developed by adding tetramethylbenzidine (TMB) substrate (Cell Signalling Technology) and the reaction was stopped by addition of 0.16M sulphuric acid. The absorbance recorded at 450 nm (OD450) and the end-point antibody titers were expressed as the reciprocals of the final detectable dilution. The cut-off was defined as the mean of control samples plus 3 standard deviations.

### Antibody-dependent cellular cytotoxicity (ADCC) assay

The ADCC activity of the serum antibodies was measured with the Promega ADCC Reporter Bioassay, according to the manufacturer’s instructions. Briefly, 50,000 MDCK cells were seeded into the wells of a white clear-bottom 96-well plate and grown overnight. Cells were then infected with 5 MOI of influenza A/Netherlands/602/09 (H1N1) for 24 h. Serum samples were heat-inactivated for 30 minutes at 56°C, serially diluted, and added to the infected cells. Mouse FcγRIV effector cells (Promega) were then added at 100,000 cells/well. After incubation at 37°C/5% CO2 for 5 hours, Bio-Glo™ luciferase assay substrate (Promega) was then added. Luminescence values were read in relative luminescence units (RLU) after 5 minutes. ADCC activity is expressed as fold induction, relative to a “no antibody” control.

### Multiplex ELISA

Spleens were collected from vaccinated animals and stimulated with 5 μg/ml of each of the selected peptides (TYQRTRALV and ASNENMETM) after homogenization [47]. Following a 48-hour incubation, the supernatant was collected for downstream measurements. NALT was collected according to the protocol described in previous sections and cultured ex vivo. The supernatant was collected for downstream measurements. Cytokine secretion in the supernatants from both tissues were measured using a ProcartaPlex Multiplex Immunoassay kit (Life Technologies). The plates were read on a Luminex 200 system (MilliporeSigma). Data analysis was performed using MILLIPLEX Analyst version 5.1 software for determining pg/ml of each cytokine.

### Proliferation assay

The proliferation assay was performed as described previously [48], with modifications to adapt to mouse lung samples. Briefly, the lungs were collected and digested into a single-cell suspension with a lung dissociation kit according to manufacturer’s instructions (Miltenyi Biotec, 130-095-927). After passing each sample through a 70 μm cell strainer, the cells were labelled using the CellTrace™ Violet Cell Proliferation Kit (Invitrogen, C34571) at a concentration of 1.0 × 106 cells/mL for 20 min at room temperature, protected from light. We then added five times the original staining volume of RPMI medium (10% FBS) to the cells for 5 min at RT to quench unbound dyes. Cells were then cultured at a concentration of 250,000 cells per well in the presence of 1 μg/mL NP peptide pool (TYQRTRALV and ASNENMETM). Stimulation with an equal concentration of DMSO in PBS was performed as a negative control. After incubation at 37 °C, 5% CO2, and 95% humidity for 72 hours, cells were washed and stained with Live/dead dye (ThermoFisher L34971), anti-CD3 (BD 564010), anti-CD4 (BD 553051), and anti-CD8 (BD 553030) for analysis by flow cytometry on FACSymphoney A1.

### FTY720 administration and lung flow cytometry

FTY720 (Sigma SML0700) was dissolved in 0.9% NaCl and administered intraperitoneally (i.p.) daily at 1 mg/kg body weight, starting three days before challenge, and continued for five more days after challenge till necropsy. An equal volume of PBS was administered as control.

To validate the FTY720 treatment, whole blood was collected in EDTA blood tubes (BD 0265732) at necropsy. Red blood cells (RBC) were lysed with RBC lysis buffer (ThermoFisher 501129743) and quenched with RPMI (10% FBS) according to manufacturer’s instructions. After washing with media, the cells were stained with Live/dead dye (ThermoFisher L34971), anti-CD45 (BD 561874), anti-CD3 (BD 563123), anti-CD4 (BD 552775), and anti-CD8 (BD 566409). The excess antibodies were washed away with FACS buffer and cells were fixed with fixation buffer (BD 554655) for 30 minutes at 4°C prior to analysis by flow cytometry (FACSymphoney A1) the next day.

At necropsy, the lungs were collected and digested into a single-cell suspension with a lung dissociation kit according to manufacturer’s instructions (Miltenyi Biotec, 130-095-927). After passing each sample through a 70 μm cell strainer and washed with PBS, the cells were stained with Live/dead dye (ThermoFisher L34971), anti-CD45 (BD 561874), anti-CD4 (BD 552775), anti-CD8 (BD 566409), anti-CD69 (562920), and anti-CD103 (BD 565529). The excess antibodies were washed away with FACS buffer and cells were fixed with fixation buffer (BD 554655) for 30 minutes at 4°C prior to analysis by flow cytometry (FACSymphoney A1) the next day.

### Quantification and statistical Analysis

Statistical analyses were conducted using Mann–Whitney test or one-way ANOVA when appropriate. Bonferroni post-tests were used to adjust for multiple comparisons between different test groups. All statistical analyses were performed using GraphPad Prism 9 software.

## Results

### Intranasal administration of an adenovirus-based vaccine afforded better cross-subtype protection against lethal influenza challenges than intramuscular administration

**Figure 1.**
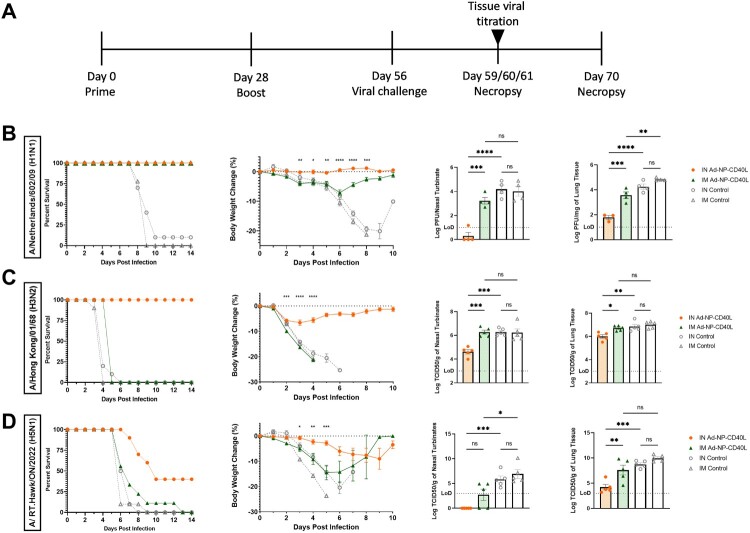
Intranasal immunization provides superior cross-subtype protection against lethal influenza challenges compared to intramuscular immunization. (a) Schematic diagram of the immunization, viral challenge, and necropsy timeline for all three strains of IAV challenge. BALB/c mice were IN or IM administered Ad-NP-CD40L or Ad-Empty as controls with a prime/boost regimen, followed by an intranasal challenge of (b) 1000 PFU of A/Netherlands/602/09 (H1N1), (c) 3.85 × 10^5^ PFU of A/Hong Kong/01/68 (H3N2), or (d) 10 PFU of A/ RT.Hawk/ON/2022 (H5N1). (Left to right) Survival (n = 10), change in body weight (n = 10) (unpaired T-test comparisons between intranasally (IN) and intramuscularly (IM) vaccinated Ad-NP-CD40L groups are shown), viral titration from infected nasal turbinates (n = 3 or 4) and lungs (n = 3 or 4) are shown. (one-way ANOVA with Bonferroni post-test). n.s. = not significant, *p < 0.05, **p < 0.01, ***p < 0.001, ****p < 0.0001. Limit of detection (LoD).

To evaluate the efficacy of Ad-NP-CD40L, BALB/c mice received 10^9^ PFU of the recombinant adenovirus in a prime and boost regimen four weeks apart through IN or IM administration. Four weeks post-boost, mice were challenged with a lethal dose of either H1N1, H3N2, or a HPAI H5N1 strain (Figure 1(a)). Following the H1N1 challenge, while both IN and IM vaccination provided complete protection with 100% survival, we did observe differences in morbidity and viral burden in both the upper respiratory tract (URT) and the lower respiratory tract (LRT). Specifically, while the IN vaccinated group had no weight loss upon challenge, significant bodyweight loss was observed in the IM group. This result is in agreement with the viral titration data, where IN vaccinated mice had lower viral burden than the IM group in both the nasal turbinate and lung tissues (Figure 1(b)). In vaccinated animals challenged with H3N2 (Figure 1(c)), the difference between IN and IM immunization was further accentuated. While IN administration provided complete protection (100% survival rate) against the lethal challenge with minimal weight loss (approx. 5%), IM vaccinated animals had similar disease progression to that of the control groups. Moreover, viral titration of infected tissues showed that only IN and not IM administration significantly reduced viral burden in both the nasal turbinates and the lungs (Figure 1(c)). In short, it is clear that for type A influenza virus (IAV) subtypes currently in circulation (H1N1 and H3N2), IN administration of NP vaccine provides superior protection than IM.

Given the heightening concerns with HPAI, we tested vaccine efficacy against a highly virulent avian strain that was recently isolated from a red-tailed hawk in Ontario, Canada [49]. The H5N1 strain, A/RT.Hawk/ON/2022, displayed high virulence and lethality in three commonly used mammalian models for influenza disease studies. More importantly, it was shown that it can be efficiently transmitted by direct contact between ferrets. As demonstrated by Kobasa et al., the LD50 for this strain in BALB/c model was <1 PFU [49]. As such a low challenge dosage is impractical for inoculation, animals were challenged with 10 PFU of the virus to maintain consistency across the animals with respect to inoculation dosage. Compared to the control groups at 0% survival, IN administered animals had a 40% survival and a significantly delayed disease progression, while the IM vaccination had no effect on improving the survival rate when compared to the controls (Figure 1(d)). The IN vaccinated animals also experienced significantly less weight loss on days 3, 4, and 5 post-challenge compared to the IM group. Lung viral titration results are in accordance with survival and weight loss data, with the IN group having significantly reduced viral burden. Although the difference in nasal turbinate viral burdens is not significant between IN and IM, IN administration resulted in a greater reduction of viral burden compared to the control groups. These results indicate that IN administration remains superior to IM administration with respect to protection from morbidity and mortality, when challenged with the HPAI strain. Interestingly, despite the vaccine NP sequence being highly similar to the NP from challenge virus strain, HPAI A/RT.Hawk/ON/2022 (Supp Figure 1), the IN vaccination failed to provide full protection, suggesting that other factors could be at play (see discussion).

### Intranasal vaccination induced strong recall immune responses in the NALT

**Figure 2.**
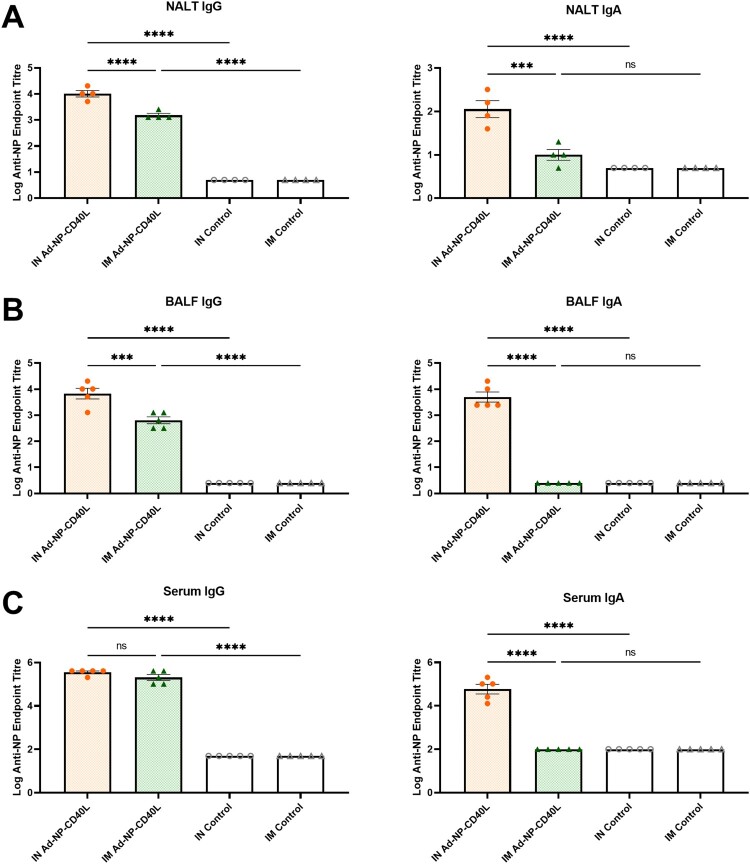
Intranasal immunization elicits stronger NP-specific IgG and IgA antibody responses along the respiratory tract compared to intramuscular immunization. (a) Nasal-associated lymphoid tissue (NALT) was collected on Day 61, 5 days after an intranasal challenge of 1000 PFU of influenza A/Netherlands/602/09 (H1N1). After 24 hours of ex vivo culture, the supernatant was collected to determine anti-NP IgG and IgA endpoint titers (n = 4). (b) Bronchoalveolar lavage fluid (BALF) was collected 4 weeks post-boost (Day 56) to determine anti-NP IgG and IgA endpoint titers (n = 5). (c) Serum from vaccinated mice were collected 4 weeks post-boost (Day 56) to determine anti-NP IgG and IgA endpoint titers (n = 5) (one-way ANOVA with Bonferroni post-test). n.s. = not significant, ***p < 0.001, ****p < 0.0001.

Having observed enhanced protection following IN administration, we next investigated the mucosal and systemic humoral responses by determining NP-specific antibody levels in the respiratory tract and serum. As a part of the URT, the NALT is the initial site of recognition and elimination of inhaled pathogens. In NALTs collected post-challenge, we observed significantly higher levels of NP-specific IgG and IgA in the IN group compared to the IM group (Figure 2(a)). A similar trend was observed in the post-boost bronchoalveolar lavage fluid (BALF), which is indicative of the humoral responses in the LRT (Figure 2(b)). Interestingly, IN and IM administration induced similar levels of systemic humoral responses, as detected by enzyme-linked immunosorbent assay (ELISA) in the sera of post-boost vaccinated animals (Figure 2(c)). Notably, only IN administration induced significant levels of NP-specific IgA in the NALT, BALF, and serum (Figure 2(a–c)).

### Both administration routes induced balanced Th1/Th2 systemic responses with IM inducing enhanced antibody effector functions

**Figure 3.**
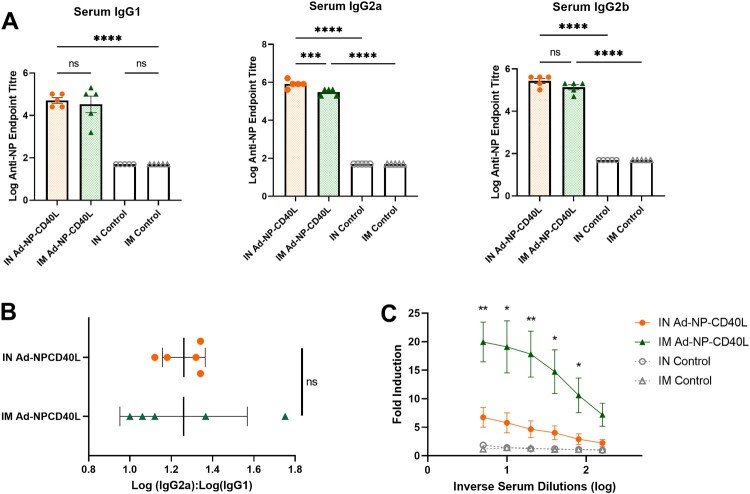
Intranasal and intramuscular immunization both elicit a balanced systemic Th1/Th2 immune response. (a) Serum from vaccinated mice were collected 4 weeks post-boost (Day 56) to determine log of anti-NP IgG1, IgG2a, and IgG2b endpoint titers (n = 5). Data shown is mean ± SEM. (one-way ANOVA with Bonferroni post-test) ns = not significant, ***p < 0.001, ****p < 0.0001. (b) NP-specific serum IgG2a:IgG1 ratio indicating Th2 – or Th1-biased nature of the immune response (n = 5). Data shown is mean ± SEM. (unpaired T-test) ns = not significant (c) Post-boost serum was used to determine the antibody-dependent cellular cytotoxicity against A/Netherlands/602/09 (H1N1) (n = 4). Data shown is mean ± SEM. (unpaired T-test comparisons between IN and IM vaccinated groups are shown) *p < 0.05, **p < 0.01.

Given the comparable anti-NP IgG levels in the sera, we further determined the IgG subtypes induced by the two routes of administration. IN administration induced similar levels of IgG1 and IgG2b relative to IM administration, but IN elicited a slightly higher level of IgG2a (Figure 3(a)). To characterize the type of immune response, we calculated IgG2a:IgG1 ratio. Both routes had similar ratios of just above 1.0, which indicated a balanced Th1/Th2 with a slight Th1-skew (Figure 3(b)).

As expected, antibodies induced by NP, an interior viral protein, have no neutralizing activities targeting viral entry (not shown). However, it has been shown, both *in vivo* and *in vitro*, that NP is expressed on the surface of infected cells despite being an internal viral protein [50, 51]. Therefore, we next investigated the antibody-dependent cellular cytotoxicity (ADCC) effector functions of the serum antibodies. While IN administration induced significant levels of ADCC antibodies compared to the controls, IM immunization group displayed a higher level of ADCC activity compared to the IN group (Figure 3(c)). We did not observe any ADCC activity in BALF likely due to the lower antibody concentrations when compared to serum samples and/or the sensitivity of the assay.

### Distinct systemic and mucosal cytokine profiles between the two routes of administration

**Figure 4.**
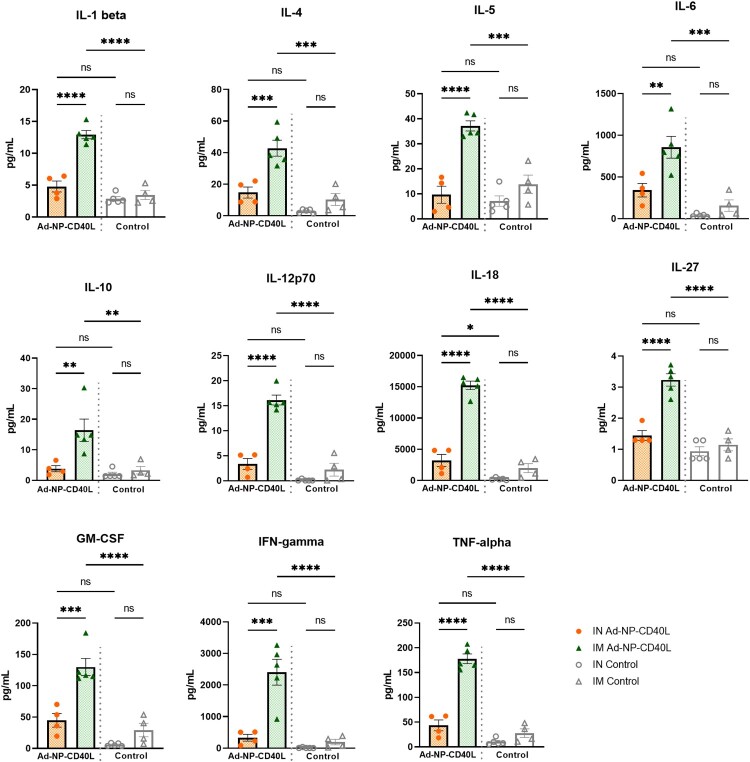
Intramuscular immunization stimulated higher cytokine responses in the spleens compared to intranasal immunization. Splenocytes were isolated 4 weeks post-boost (Day 56) and incubated with TYQRTRALV and ASNENMETM at 5 µg/ml each (n = 4 or 5). Following 72-hour incubation, cytokines in the supernatant were quantified with a Luminex system. Data shown is mean ± SEM. (one-way ANOVA with Bonferroni post-test) ns = not significant, *p < 0.05, **p < 0.01, ***p < 0.001, ****p < 0.0001.

We next investigated cytokine production following vaccination by IN or IM routes. To evaluate systemic responses, splenocytes from vaccinated animals were stimulated with immunodominant NP peptides prior to the quantification of secreted cytokines. IM administration induced about 2–6 folds higher cytokine production upon stimulation compared to the IN group, including Th1, Th2, and Treg cytokines (Figure 4). Notably, compared to their respective controls, Th1 cytokines such as IFN gamma, TNF-alpha, and Interleukin(IL)−18 were produced at higher levels (about 6–10 folds higher than the control) when compared to Th2 cytokines (about 2–3 folds higher), such as IL-4, IL-5, and IL-10. Similarly, IN administration also induced significant levels of Th1 cytokines compared to its respective control, such as IFN gamma, TNF-alpha, and IL-18 (about 4–24 folds higher), although at a lower level than IM administration. The spleen cytokine profile is consistent with the systemic antibody subtypes, both administration routes demonstrating the Th1-skewed nature of the induced response.

**Figure 5.**
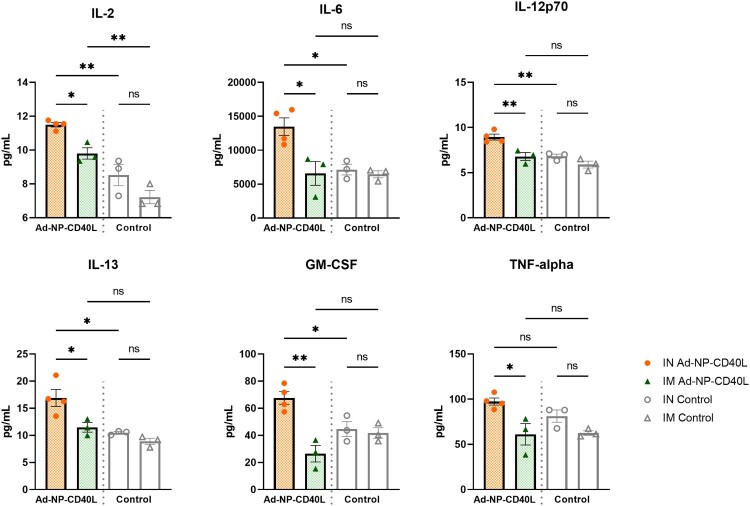
Upon infection, intranasal immunization induced stronger cytokine responses in the nasal associated lymphoid tissue (NALT) compared to intramuscular immunization. NALTs were collected on Day 61, 5 days after intranasal challenge of 1000 PFU of influenza A/Netherlands/602/09 (H1N1). After 24 hours of ex vivo culture, a Luminex system was used to quantify cytokines in the supernatant (n = 4). Data shown is mean ± SEM. (one-way ANOVA with Bonferroni post-test) ns = not significant, *p < 0.05, **p < 0.01.

Having examined the systemic cytokine production in the splenocytes, we next determined cytokine responses in the local mucosal tissues using the same cytokine panel. As shown in Figure 5, IN immunization induced higher levels of cytokine when compared to IM immunization in the NALT by about 1–2.5 folds. Furthermore, IN vaccination elevated the levels of Th1 cytokines such as TNF-alpha, IL-12p70 and GM-CSF when compared to IM administration. While like other secondary lymphoid organs, the NALT may contain a small number of circulating lymphocytes within the tissue. It is of note, however, that a stimulated NALT is predominantly composed of non-circulating activated B cells and CD4+ T cells that help B cells to respond [52]. Other than the cytokines shown in Figure 5, we did not find any other significantly elevated cytokines in the NALT compared to the controls. It is to be noted that the NALT is a much smaller tissue compared to the spleen, made of approximately 10^5^ lymphocytes [53]. Therefore, the cytokine production levels of the two tissues cannot be directly compared. Nonetheless, these results collectively indicate that IN immunization induces a more robust mucosal immunity with a cell-mediated response profile that is distinct from that of the IM response at the local mucosal tissues.

### More robust antigen-specific pulmonary T cell responses were detected after IN vaccination

**Figure 6.**
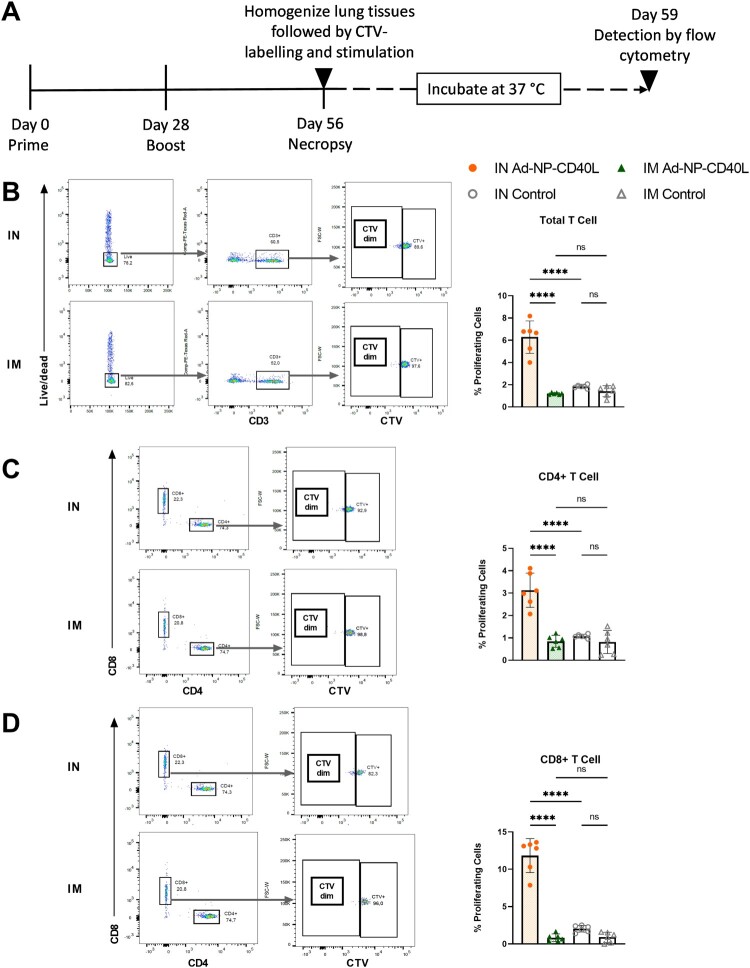
Intranasal immunization induces robust antigen-specific T cell proliferation in the lungs compared to intramuscular immunization. (a) Schematic diagram of the immunization, necropsy, and proliferation assay timeline. BALB/c mice were intranasally (IN) or intramuscularly (IM) administered Ad-NP-CD40L or Ad-Empty (Control) with a prime/boost regimen on Day 0 and Day 28 and were necropsied on Day 56. The lung tissues were digested, homogenized, and stained using CellTrace® Violet (CTV). After quenching and washing, the lung cells were stimulated with immunogenic peptides from NP. Following 72-hour incubation, frequency of proliferating cells was measured by flow cytometry. Graphs showing representative gating strategies and raw frequency of proliferating cells in (b) total T cells, (c) CD4+, (d) CD8 + populations. (one-way ANOVA with Bonferroni post-test) Data shown is mean ± SEM. ns = not significant, ****p < 0.0001.

Having observed a distinction in the cytokine profiles among the systemic and local mucosal immune responses following the two different routes of administration, we next investigated antigen-specific T cell proliferation in the lungs. The lung tissues from vaccinated mice were homogenized and stained using CellTrace® Violet (CTV), a fluorescent dye that diminishes as the labelled cell proliferates. Proliferation was thus measured as the percentage of cells with diminished CTV stain (Figure 6(a)). The percentage of proliferating cells were measured for each of the CD3+, CD4+, and CD8+ T cell populations. In all three T cell populations, IN vaccinated mice had more antigen-specific T cell expansion when compared to the IM vaccinated group (Figure 6(b–d)). Notably, the fold increase in proliferating CD8+ T cells was the highest among the T-cell populations studied. These results indicate that IN immunization induces more proliferative antigen-specific T cells in the lungs than IM vaccination, which may lead to a faster recall response upon infection.

### Blocking lymphocytes egression had no impact on IN vaccination efficacy

**Figure 7.**
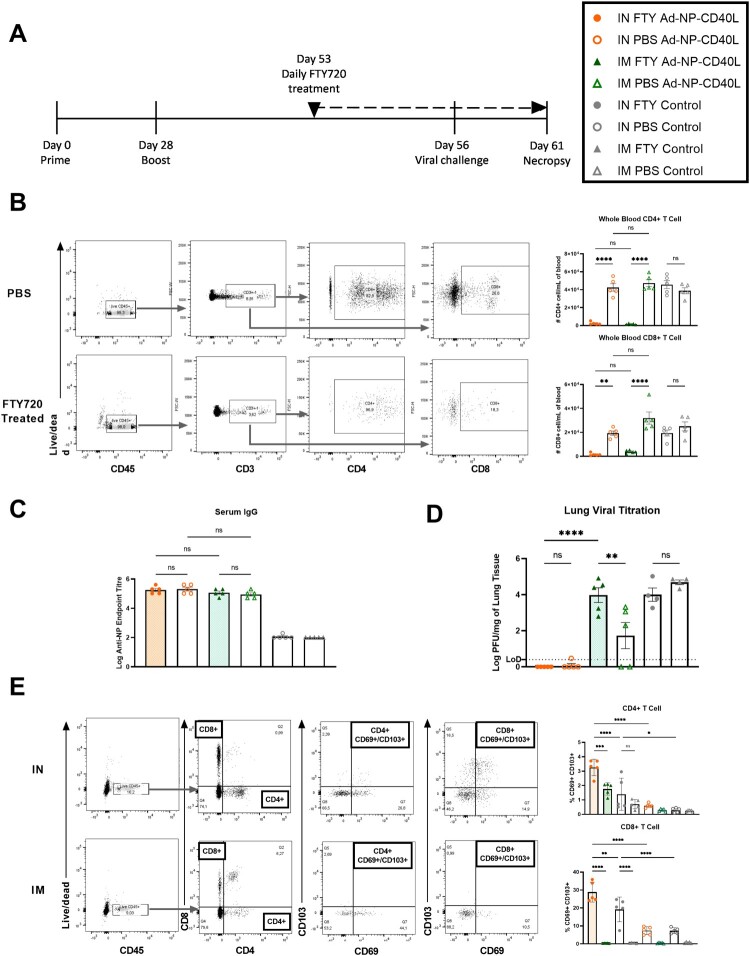
Intranasal immunization provided better protection without circulating lymphocytes than intramuscular immunization. (a) Schematic diagram of the immunization, FTY720 treatment, viral challenge, and necropsy timeline. BALB/c mice were intranasally (IN) or intramuscularly (IM) administered Ad-NP-CD40L or Ad-Empty (Control) with a prime/boost regimen on Day 0 and Day 28, followed by daily FTY720 or PBS intraperitoneal injections starting on Day 53 for 9 days. The animals were challenged with 1000 PFU of influenza A/Netherlands/602/09 (H1N1) on Day 56 and necropsied on Day 61. (b) The number of CD4 + and CD8 + cells were quantified in the blood via flow cytometry to validate the FTY720 treatment (n = 5). Graph showing representative gating strategies. Data shown is mean ± SEM. (c) Serum was collected at necropsy to determine log of anti-NP IgG endpoint titer (n = 5). (d) Viral titre from infected lungs (n = 5) collected 5 days post-challenge were determined by plaque assay. Data shown is mean ± SEM. (E) The frequency of CD69+/ CD103 + cells in the CD4 + and CD8 + populations in the lungs were determined by flow cytometry (n = 5). Graph showing representative gating strategies. Data shown is mean ± SEM. (one-way ANOVA with Bonferroni post-test) ns = not significant, **p < 0.01, ***p < 0.001, ****p < 0.0001.

As elevated immune responses were detected in the respiratory tract following IN immunization, our next aim was to test whether it could confer protection in the absence of circulating lymphocytes. We utilized the drug FTY720, a sphingosine-1-phosphate receptor 1 agonist, which prevents lymphocyte egress from lymphoid tissues and bone marrow into circulation. It is an approved oral treatment for relapsing forms of multiple sclerosis [54, 55]. IN or IM vaccinated mice were intraperitoneally (IP) administered with FTY720 or PBS daily, starting on Day 53 for 9 days up till necropsy. The animals were then challenged with H1N1 on Day 56 and necropsied on Day 61 (Figure 7(a)).

The experiment was designed to ensure effective blocking of lymphocyte circulation, in order to dissect the protective roles of antibody responses and T cells. We first validated the effectiveness of the FTY720 treatment by quantifying the number of T cells in whole blood via flow cytometry. Overall, there was a drastic decrease in the number of both CD4 + and CD8+ T cells in the blood after FTY720 treatment compared to the PBS groups. There were no significant differences between PBS treatment groups of IN and IM Ad-NP-CD40L vaccinated animals (Figure 7(b)). It is of note that no significant differences were detected in the anti-NP antibody levels between the treatment and non-treatment groups (Figure 7(c)). This is to be expected since B cells and antibody production do not depend on circulating lymphocytes. Therefore, the humoral response is not a major contributor to the differences in protection that are observed.

Importantly, as evident in Figure 7(d), the FTY720 treatment did not affect the protection conferred by IN vaccination. Irrespective of the treatment, the lung viral load was undetectable by plaque assay. In contrast, FTY720 treatment resulted in a significant increase in viral load in the IM group. To further characterize the CD4 + and CD8+ T cells in the lungs, they were stained for the co-expression of two tissue resident cells (TRM) markers, CD69 and CD103. As shown in Figure 7(e), IN vaccination induced a higher percentage of CD69+/CD103 + cells than the IM group among Ad-NP-CD40L vaccinated animals. Interestingly, an elevation of CD69+/CD103 + cells in the IN control was also observed. This is likely due to adaptive immune responses triggered by epitopes present in the adenoviral vector, as part of its adjuvating effects as a vaccine vector [56]. In addition, compared to their no-treatment PBS controls, the percentages of CD69+/CD103 + cells were increased by the FTY720 treatment. This effect was most prominent in the IN vaccinated Ad-NP-CD40L group. Importantly, however, the number of CD69+/CD103 + cells in the IN Ad-NP-CD40L vaccinated mice was significantly higher than the IN control group. Taken together, these results demonstrate that IM vaccination relies on the circulating lymphocytes for protection, whereas the IN vaccination relies on local tissue responses that display characteristics of tissue resident cells and can provide full protection in the absence of circulating lymphocytes.

## Discussion

The ever-evolving nature of the influenza surface glycoproteins requires annual update to the influenza vaccines. While the development of HA-based universal vaccines has been vigorously pursued [57], substantial interests remain in exploring the potential of more conserved antigens, such as NP [58, 59]. As aforementioned, a variety of vaccine platforms have been explored to deliver NP [11–25]. Most studies supported NP as a candidate target, with several studies demonstrating that mucosal delivery elicits a more robust protection compared to other routes of administration. These studies have employed various constructs and platforms including mRNA [33], polyvalent virosome [60], and co-administration of multiple recombinant vectors carrying different viral proteins [11, 12, 34, 61]. Our current study differs from these previous studies in two main aspects, which are the nature of the NP antigen and the head-to-head comparison between IN and IM administrations. Specifically, we utilized an adenovirus vector-based vaccine expressing NP fused with the ectodomain of CD40L, with CD40L functioning as both a targeting ligand and a molecular adjuvant. As shown in our previous work, the subcutaneous (SC) delivery of this vaccine did not provide full protection against lethal influenza challenges, with only 50-70% survival [43], suggesting the need of improvement. We aimed to answer two questions: first, would IN or IM delivery improve the efficacy of Ad-NP-CD40L? Second, if so, what underlying mechanisms mediate differences in protection induced by IN and IM administration?

To assess the breadth of protection, BALB/c mice were challenged with H1N1, H3N2, and a newly identified HPAI H5N1 strain. Irrespective of the challenge strain, IN administration provided significantly better protection than IM administration, an observation largely in agreement with other comparison studies [11, 12, 33, 34, 60, 61]. Notably, while both IN and IM vaccination provided 100% survival against H1N1 challenge, IM administration failed to prevent body weight loss and had significantly higher viral burden in the URT and LRT (Figure 1(b)). The difference in protection between the two routes was more prominent following H3N2 challenge, where IM administration failed to provide any protection relative to negative controls (Figure 1(c)). These data indicate that IN vaccination is superior to both IM vaccination, as found in this study (Figure 1), and SC vaccination, as observed in our previous investigation [43]. In the challenge against the HPAI H5N1 strain (A/RT.Hawk/ON/2022), IN vaccination provided partial protection, while IM administration afforded no protection (Figure 1(d)). This HPAI strain was recently isolated from a red-tailed hawk and was found to have high pathogenicity in mice, with a LD50 of <1 PFU [49]. It is to be noted that the NP sequence in this H5N1 strain is 99.3% identical to that of the vaccine NP sequence (Supp Figure 1). Therefore, the partial protection is not only due to presence of sequence conservation, but more importantly the local immune responses, as evident by the lack of protection in IM group despite the sequence conservation. While investigations are ongoing towards better understanding of the remarkable virulence of A/RT.Hawk/ON/2022 in mammalian animal models, our observations support the notion that IN vaccination of Ad-NP-CD40L provides superior cross-protection when compared to IM vaccination (Figure 1(d)). The more virulent nature of H3N2 and H5N1 has also been reported by others that they tend to have a faster disease progression than H1N1, which could lead to more severe disease outcomes if the infections are not contained in the early phase [62–64]. However, it is also likely that differences in the lethality of challenge dosage used for the strains could be a reason for the varying degree of survival conferred by the two routes of vaccinations among the different influenza strains. Regardless, although both vaccination strategies provided protection against lethality, other parameters to measure disease progression of the H1N1 infection, such as weight and viral titer, were consistent with the trend observed in the challenge experiments with the other two influenza strains (Figure 1). Results from this study and others [11, 12, 33, 34, 60, 61] have collectively reinforced the notion that IN vaccination induces more effective protection against wider range of viral strains, while the incorporation of the bifunctional CD40L ectodomain into antigens could also induce a broader immune response against a range of virus subtypes [43, 65].

Interestingly, despite IN administration providing better protection, both routes of administration induce robust serum antibody responses, with a slight Th1-skew (Figures 2 & 3). However, IM administration induced markedly stronger antibody effector function activity (ADCC) (Figure 3(c)) and antiviral cytokine responses in the spleen (Figure 4). In the absence of neutralizing antibodies, such as responses induced by NP, such defence mechanisms could play important roles [32, 66, 67]. However, as per our observation, such superior serum humoral responses are not sufficient to afford complete protection. We also noted a recent study by Vanderven *et al.*, where it was demonstrated that *in vitro* Fc receptor-binding anti-NP monoclonal antibodies are not sufficient to completely protect against challenge upon passive transfer [68]. Given that IN vaccination primarily induces local responses, ADCC and other effector functions at these mucosal sites are yet to be studied in depth, with the functional roles at mucosal site being largely unknown [69]. While we were unable to detect ADCC activity in the BALF (data not shown), our observations clearly indicate that a strong mucosal response is needed in addition to systemic responses to afford superior protection from challenge with influenza A. (Figure 6). These observations are largely in agreement with others, in particular the role of pulmonary T cell responses [61, 70].

One of the less studied lymphoid tissues in influenza vaccine research is the NALT, which is the initial site of immune recognition and elimination of inhaled pathogens in the URT [71]. In our study, strong recall antigen-specific IgA and IgG were only observed in the NALT from IN-vaccinated animals upon viral infections (Figure 2(a)). Moreover, IN vaccination also induced significantly stronger cytokine responses the NALT (Figure 5). Notably, IL-6, markedly elevated in IN vaccinated animals, is known to be a key cytokine promoting the differentiation and proliferation of plasma cell precursors and instigates the development of IgA antibodies [72], supporting the elevated IgA expression levels detected at the mucosal sites (Figure 2(a)). IL-2, previously linked to restoring mucosal immunity in aged mice, was also detected significantly higher in the NALT [73]. Notably, the small size of NALT with low number of lymphocytes in the NALTs required *ex vivo* culturing following challenge. As shown in Figures 2 & 5, NALTs from IN and IM groups were compared under the same collection timeline and culture conditions. We found IN immunization induced higher mucosal antibodies and cytokines (Figures 2 & 5), which is in agreement with tissues collected pre-challenge. These findings at the NALT are consistent with the protective responses detected in the BALF and lungs, supporting the observation of potent mucosal immunity at both URT and LRT induced by Ad-NP-CD40L IN vaccination.

Lastly, we interrogated the protective roles of circulating and resident lymphocytes. We found that blocking lymphocytes egression had minimal effect on the effectiveness of IN vaccination, while in contrast, increased viral burden was observed following IM immunization. These results are consistent with a study with a different experimental design, which was the IN co-administration of multiple recombinant vectors carrying HA, NP, and IL-1β [12], while ours contains only NP fused with a bifunctional CD40L. Furthermore, our observation was also supported by the increased frequency of resident CD69+/CD103 + cells in the CD4 and CD8 T cell populations in the lungs (Figure 7). It should be noted that multiple assays employed in this study, including cytokine quantification (Figures 4 & 5), proliferation assay (Figure 6), and the blocking circulation of lymphocytes experiment (Figure 7), allowed us to compare and dissect cellular responses induced by IN and IM administrations. Other assays such as intracellular cytokine staining and activation-induced marker assays would also have added value for future studies.

Given that the respiratory tract is the natural site of infection for adenovirus, the adenoviral vector is a desirable platform for mucosal immunization as demonstrated by various clinical studies [74–76]. During natural infection with adenovirus, pro-inflammatory cytokines, such as IL-6 and TNF-alpha, are released. However, data on the profile of cytokines released in response to mucosal vaccinations are limited [77]. A challenge faced by adenovirus-based vector vaccines is pre-existing immunity, which could weaken the efficacy of some vectors with widespread serotypes [78]. However, several approaches have been explored with promising findings as reviewed by Zhang *et al* [79]. One of these strategies is to induce immunity through the nasal delivery to overcome pre-existing immunity [11, 80, 81]. Other approaches have also proven to be promising, such as utilizing nonhuman or less common serotypes [82, 83], modifying the surface of the viral particle [84, 85], and employing heterologous prime-boost regimens [86]. Thus, in addition to inducing a protective immune response, IN immunization could also overcome issues with pre-existing immunity to the vector, when compared to IM administration.

In conclusion, we have demonstrated that IN delivery significantly improves the efficacy of Ad-NP-CD40L compared to systemic immunization, inducing superior protection against multiple influenza A strains. This superior protection was primarily mediated by mucosal responses, while the limited protection induced by IM vaccination stems from its lack of potent local immune responses. These findings, along with other reports with different experimental approaches [12, 61, 70], have advanced our knowledge of the NP-induced mucosal protection and may inform the design of future cross-subtype protective vaccines against influenza, particularly, those which employ highly conserved interior viral proteins.

## Supplementary Material

Supp Fig 3.tif

supp 2 fold change.tif

supp 1 vaccine design.tif

## Data Availability

Data have been deposited in the Government of Canada Centralized repertoire and would be available upon request after publications.
