# Supplementary figures and images for: Dissecting immunological mechanisms underlying influenza viral nucleoprotein-induced mucosal immunity against diverse viral strains
Source: Emerg Microbes Infect. 2024 Nov 7;13(1):2427792. doi: 10.1080/22221751.2024.2427792 (PMC11583363; doi:10.1080/22221751.2024.2427792)

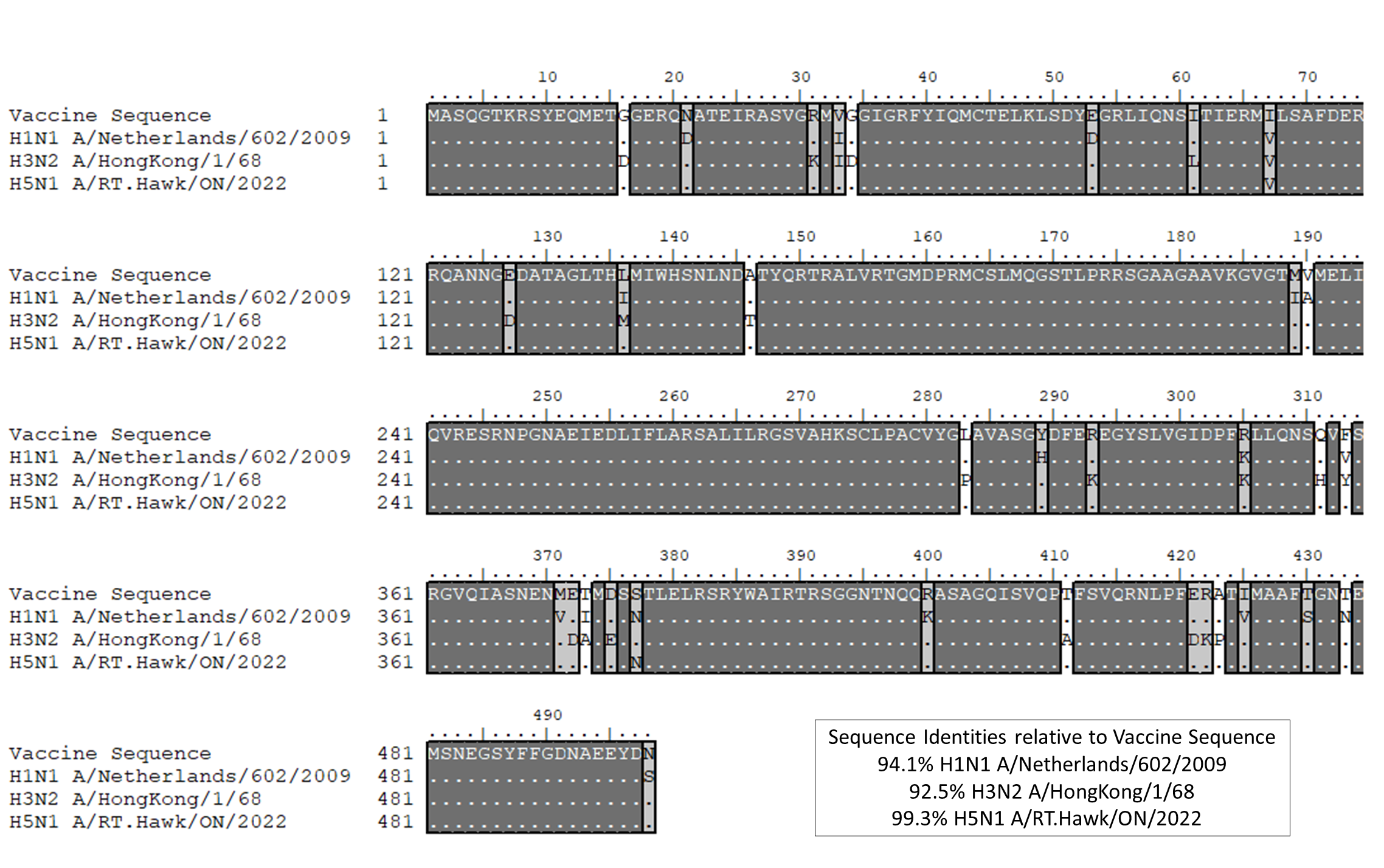

Supplement: Supp Fig 3.tif [file TEMI_A_2427792_SM4985.tif]

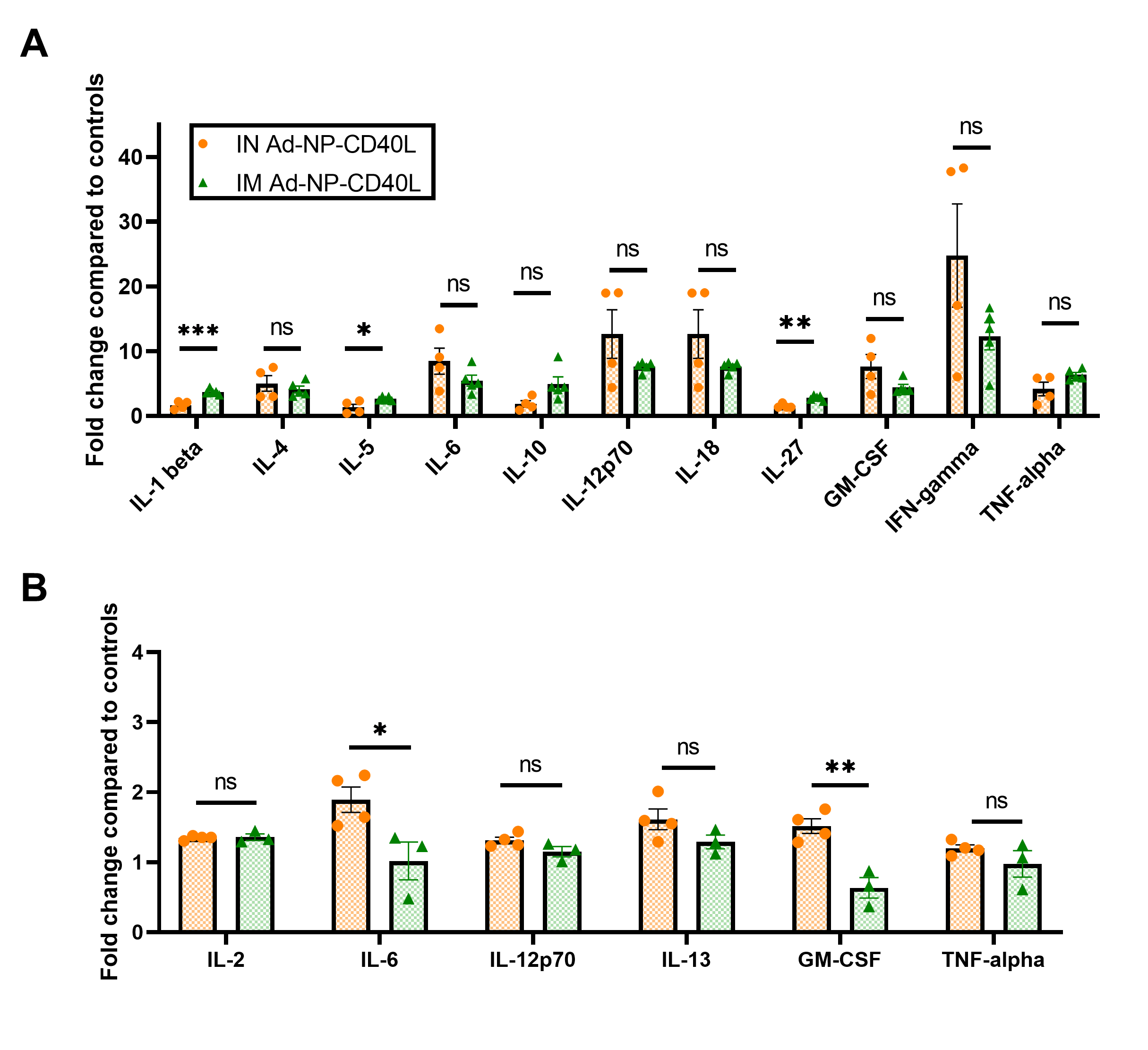

Supplement: supp 2 fold change.tif [file TEMI_A_2427792_SM4984.tif]

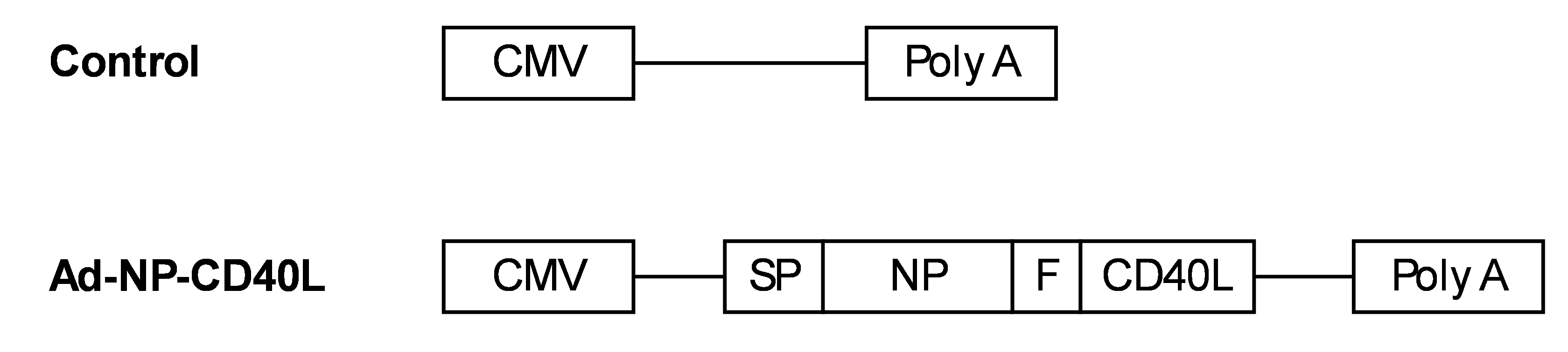

Supplement: supp 1 vaccine design.tif [file TEMI_A_2427792_SM4983.tif]
